# Urushi as a Green Component for Thermally Curable Colloidal Lignin Particles and Hydrophobic Coatings

**DOI:** 10.1021/acsmacrolett.3c00186

**Published:** 2023-05-22

**Authors:** Adrian Moreno, Ievgen Pylypchuk, Yoko Okahisa, Mika H. Sipponen

**Affiliations:** † Department of Materials and Environmental Chemistry, Stockholm University, Svante Arrhenius väg 16C, SE-106 91 Stockholm, Sweden; ‡ Laboratory of Sustainable Polymers, Department of Analytical Chemistry and Organic Chemistry, Rovira i Virgili University, Tarragona 43007, Spain; § Faculty of Fiber Science and Engineering, Kyoto Institute of Technology, Matsugasaki, Sakyo-ku, Kyoto 606-8585, Japan; ∥ Wallenberg Wood Science Center, Department of Materials and Environmental Chemistry, Stockholm University, SE-10691 Stockholm, Sweden

## Abstract

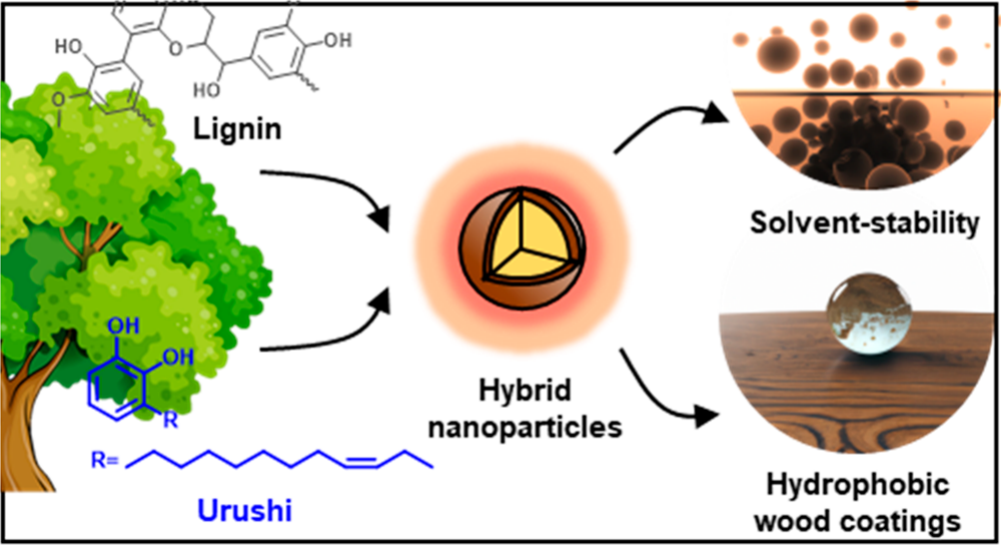

Colloidal lignin nanoparticles are promising building blocks for sustainable functional materials. However, their instability in organic solvents and aqueous alkali limits their applicability. Current stabilization methods require nonrenewable and toxic reagents or tedious workup procedures. Here we show a method to prepare hybrid nanoparticles using only natural components. Urushi, a form of black oriental lacquer, and lignin are coaggregated to form hybrid particles, with Urushi acting as a sustainable component that stabilizes the particles via hydration barrier effect and thermally triggered internal cross-linking. The weight fractions of the two components can be adjusted to achieve the desired level of stabilization. Hybrid particles with Urushi content >25 wt % undergo interparticle cross-linking that produces multifunctional hydrophobic protective coatings that improve the water resistance of wood. This approach provides a sustainable and efficient method for stabilizing lignin nanoparticles and opens up neoteric possibilities for the development of lignin-based advanced functional materials.

There is a strong need to find practical solutions to alleviate our dependence on fossil resources for the production of materials that we use in our daily lives, such as coatings, surfactants and adhesives, among others. In this sense, the past few years have witnessed a coherent development of biobased materials and chemicals.^1,2^ In fact, macromolecules from a renewable and abundant plant biomass are gaining a major role in the efforts to transition to a sustainable materials economy. Among them, the aromatic plant polymer lignin is one of the most promising biobased raw materials.^3−6^

In Nature, lignin forms part of the plant biomass, together with cellulose and hemicellulose, and its natural functions include adding strength and rigidity to the plant cell walls, enabling the transport of water and protecting from pathogens and insects. Therefore, it is not surprising that lignin is gifted with attractive properties such as high carbon content (>60 atom %), thermal stability, biodegradability, antioxidant activity, and the ability to absorb UV radiation.^7−10^ These inherent properties are strongly related to its aromatic structure and translate into a great potential in the development of advanced functional materials.^3,4^ Additionally, lignin nanoparticles (LNPs) have emerged within the past ten years, overcoming many well-known shortcomings of technical lignins such as heterogeneity and poor compatibility in polymers.^11−14^

LNPs have a well-defined spherical shape and exhibit colloidal stability due to the electrostatic repulsion forces of carboxylic acid and phenolic hydroxyl groups enriched on their surface, which prevents their aggregation in aqueous dispersions (pH 3–9) and provides a high surface area to mass ratio.^15^ This anionic surface charge has been used for the physical modification of LNPs via adsorption of positively charged compounds such as enzymes or polymers, as well as improving the compatibility within polymeric matrixes.^16−18^ Recently, photonic materials with a variety of structural colors have been achieved with LNPs.^19−21^ These developments have transformed LNPs into a thriving research field in many different applications such as biomedicine,^22,23^ water purification,^24^ composites,^25,26^ and surfactants,^27,28^ among others.^29−32^ However, when it comes to chemical functionalization of LNPs in the dispersion state, limitations associated with their dissolution in basic pH > 10 (due to the deprotonation of phenolic groups) and aggregation in acidic media pH < 2.5 (due to the neutralization of carboxylic acid groups) restricts their functionalization and potential end-uses.^6^ To overcome these limitations, our group and others have reported various methods for the stabilization of LNPs via internal cross-linking by the addition of a cross-linker during their supramolecular assembly,^33,34^ the use of oxidoreductive enzymes such as laccases,^35,36^ or by endowing LNPs with a hydration barrier derived from fatty acids.^37,38^ Among them, the use of fatty acids emerges as one of the most attractive approaches to prepare stable hybrid particles in a high yield without the use of fossil-derived cross-linking agents, which is critical for technical applications that require large quantities of lignin, such as waterbone dispersion coatings.^37^ However, the technical use of fatty acids and their derivatives can compete with food production to some extent. Hence, investigating the feasibility of nonfood components that are both sustainable and competitive for the long-term stabilization of LNPs holds great potential as a research direction.

In this work, we focused on Urushi, an oriental lacquer obtained by refining the sap of the Urushi tree (*Toxicodendron vernicifluum*) and composed mainly of urushiol, a catechol derivative with a long unsaturated hydrocarbon side chain (C_15_–C_18_) adjacent to the phenolic hydroxyl groups. The properties of Urushi are similar to other characteristic properties found in cathechol derivatives and include high water resistance, antibacterial properties, and good adhesion, which are currently attracting the attention in coating applications, such as drying oils.^39−41^ We show that by using colloidal coaggregation of Urushi with Softwood Kraft Lignin (SKL), we can create Urushi–SKL hybrid nanoparticles (UR-SKL hy-NPs) in a simple and scalable manner. The percentage of Urushi used during particle formation determines whether the resulting particles achieve stabilization through internal cross-linking (<25 wt % of Urushi) or a hydration barrier owing to the accumulation of hydrophobic chains close to the particle surface (>25 wt % of Urushi). Finally, we demonstrate a proof of concept of these particles as hydrophobic dispersion coatings that increase water resistance of wood.

Our approach to solvent-stable LNPs starts with the preparation of Urushi-SKL hybrid nanoparticles (UR-SKL hy-NPs). The preparation of the UR-SKL hy-NPs, thereafter named as hy-LNPs was performed via solvent-exchange coaggregation of SKL with Urushi (Figure 1). A binary solvent mixture of tetrahydrofuran–water at mass ratio 3:1 was selected to ensure a complete dissolution of the starting materials and hybrid particles were formed by rapid pouring of water to the initial binary solvent mixture containing SKL and Urushi.

**Figure 1:**
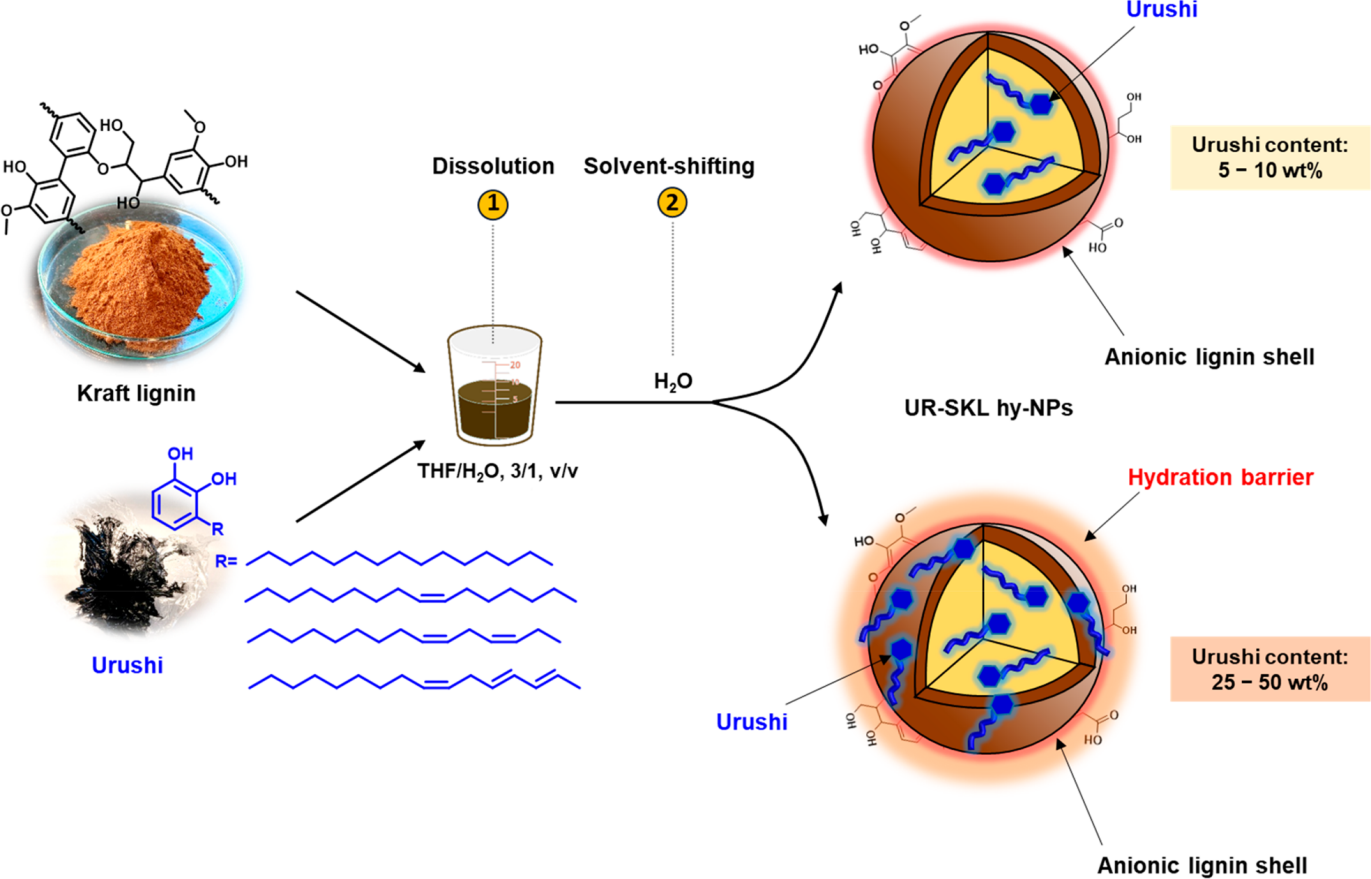
Schematic illustration of the preparation of Urushi-kraft lignin hybrid nanoparticles (UR-SKL hy-NPs) via solvent exchange coprecipitation method. The orange color in the representation of UR-SKL hy-NPs (bottom) indicates the presence of a hydration barrier produced by the exposure of the hydrophobic Urushi chains close to the surface of the particle at high concentration of Urushi (>25 wt %).

Compared to covalent synthetic procedures this simple approach presents benefits within several green chemistry principles. Regardless of the content of Urushi (5–50 wt %) colloidal stable nanoparticle dispersions were obtained in all cases with a mass yield exceeding 90% (Figure 2b). The mass content of Urushi was found to dictate the particle size. Dynamic light scattering (DLS) of colloidal dispersions showed that the particle diameter increases with the increment of urushi content (e.g., 120 nm for 10 wt % of Urushi content and 284 nm for 50 wt % of Urushi content, Figure 2a). This fact can be rationalized by the presence of urushi, with a highly hydrophobic nature, in the core of the particle together with high molecular weight lignin fractions, while the surfaces would be composed of relatively less condensed lignin units enriched with relatively more hydrophilic carboxylic acid and phenolic groups, as suggested by previous experimental works on the formation mechanism of LNPs.^15,37^ This structural model of hybrid NPs, where the surface is populated mainly by charged lignin residues, agrees with the analysis of the ζ-potential of different hy-LNPs, which were found close to each other (−28 to −32 mV, Figure 2a). Consequently, a good colloidal stabilization arises from the surface-oriented carboxylic acid and phenolic hydroxyl groups of lignin. However, a slight decrease in ζ-potential with an increase in the Urushi content can be also appreciated, and associated with structural reordering on hy-LNPs, presumably caused by the redistribution of Urushi hydrophobic chains exposed to the particle surface. This observation was further confirmed with transmission electron microscopy (TEM) imaging of the colloidal dispersions (Figure 2c,d). The TEM micrographs for hy-LNPs with 10 wt % of Urushi (hy-LNPs_10_) revealed a core–shell structure attributed to the presence of Urushi in liquid form inside the hy-LNPs (Figure 2c, red arrows), which is consistent with the case of hy-LNPs with higher content of Urushi (50 wt %, hy-LNPs_50_) and attest the high encapsulation capability of LNPs toward poorly water-soluble molecules (Figure 2d). Here it is worth to mention that aside having a core–shell structure, hy-LNPs_50_ have a a high tendency to agglomerate upon drying (Figure 2d, yellow arrows). The reason for the particles to stick together is believed to be the result of the hydrophobic Urushi chains coming together and forming clusters on the surface of the particle due to the hydrophobic effect, which is more pronounced at higher concentrations when the chains collapse during the drying process (Figure 1). In line with these findings, SEM imaging of hy-LNPs_50_, hy-LNPs_25_ and hy-LNPs_10_ (Figures 2e and S1) also support the high tendency of hy-LNPs to agglomerate upon increasing the concentration of Urushi. In addition, AFM images also revealed a dented surface of hy-LNPs_50_ in contrast to hy-LNPs_10_ (compare Figure 2f and Figure S2) and confirm the high tendency of hy-LNPs50 to collapse and aggregate as a consequence of the presence of hydrophobic Urushi chains close to the surface of the particles. As a result of having a higher amount of Urushi, hy-LNPs not only have a charged surface, but also have hydrophobic Urushi chains that act as a barrier to prevent water from entering the particles. Previously, we observed a similar effect during the creation of lignin-oleic nanoparticles.^37,38^

**Figure 2:**
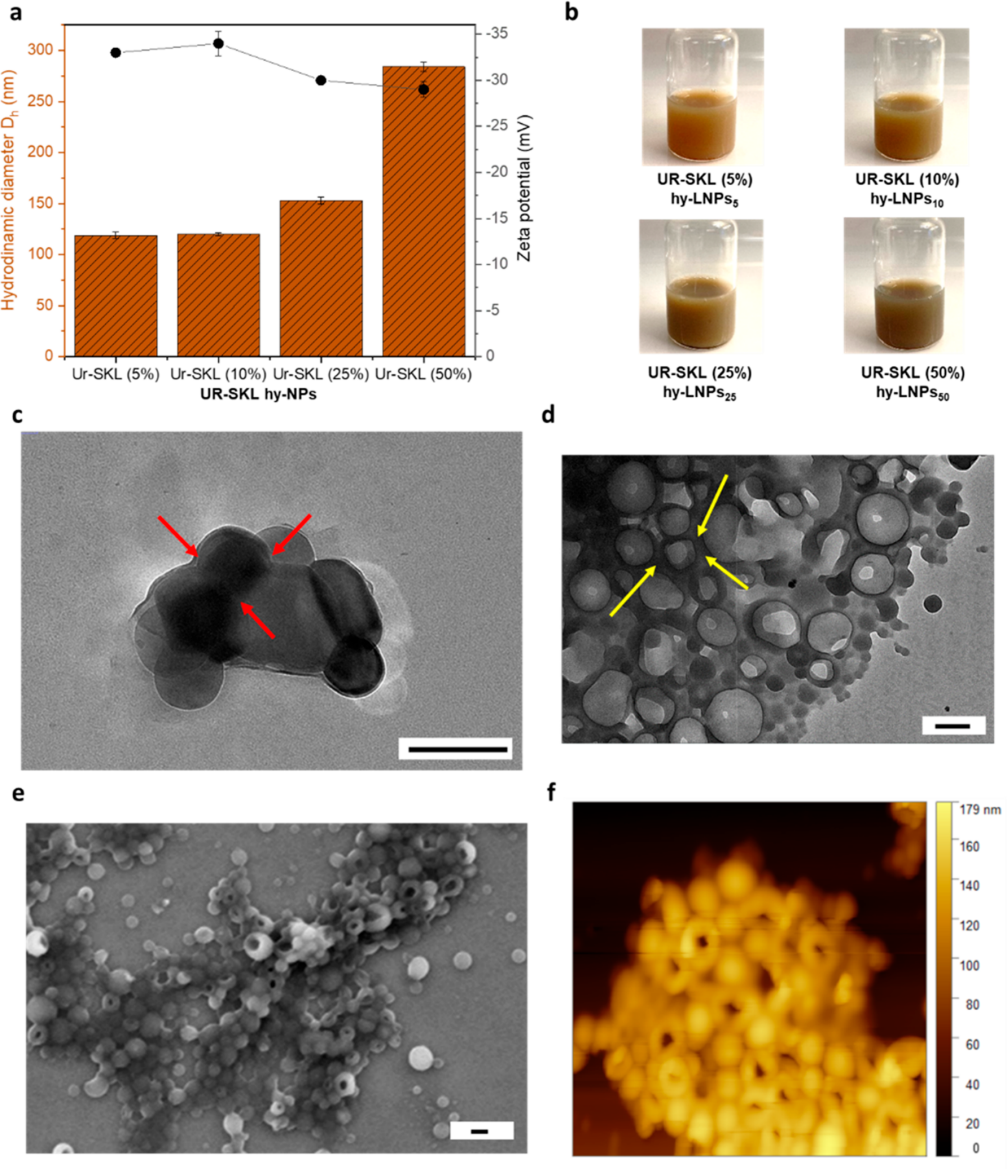
Characterization of hy-LNPs: (a) Size distribution and zeta potential of hy-LNPs. (b) Digital images hy-LNPs colloidal dispersions. Transmission electron microscopy (TEM) images of (c) hy-LNPs_10_ and (d) hy-LNPs_50_. (e) Scanning electron microscopy (SEM) image of hy-LNPs_50_. (f) AFM height images of hy-LNPs_50_. Scale bars (200 nm). The error bars in a represent ± standard deviation (SD) from the mean values (*n* = 3).

Based on our above findings, we were intrigued with the accumulation of the hydrophobic Urushi chains close to the surface of hy-LNPs would enhance colloidal stability under harsh conditions. In this sense, kinetic experiments under harsh conditions (pH > 12) were investigated for the hy-LNPs with different Urushi content (from 5 to 50 wt %) prepared in this work (Figure 3a). Time-dependent size measurements of particles with lower content of Urushi (hy-LNPs_5_, 5 wt % of Urushi and hy-LNPs_10_, 10 wt % of Urushi) revealed a significant decrease in particle size after 25 and 56 h, respectively, as a consequence of complete dissolution of lignin owing to the deprotonation of phenolic hydroxyl groups at basic pH (Figure 3a, black squares and red circles). In contrast, hy-LNPs with high Urushi content (hy-LNPs_25_, 25 wt % of Urushi and hy-LNPs_50_, 50 wt % of Urushi content) remained essentially stable with no significant changes in the particle size for more than 200 h, which should suffice for their chemical modification in dispersion state. This extraordinary stability is attributed to the presence of hydrophobic Urushi chains close to the particle surface, acting as a hydration barrier membrane that prevents the ionization of the phenolic groups. In order to support our findings, ^1^H NMR of hy-LNPs_25_ and hy-LNPs_50_ in dispersion state were also conducted (Figure S3). ^1^H NMR spectra in dispersion state of the hybrid particles revealed signals corresponding to the double bond present in Urushi, and thus support the progressive accumulation of Urushi’s hydrophobic chains close to the particle surface (Figure S3). Similar results were previously obtained with lignin oleate in which partial esterification of the hydroxyl groups of lignin change its physicochemical properties.^37^ Here it is important to highlight that supramolecular aggregation of lignin and Urushi produced hybrid particles that significantly exceeded the ∼150 h stability threshold of lignin oleate particles, being stable for more than 3 months at pH 12 without need of internal cross-linking process. The reason behind this superior stability is based on the fact that there are no chemical linkages between Urushi and lignin in the hy-LNPs presented herein, and as a consequence the stability of the particles is exclusively derived from the hydrophobic interactions of the aliphatic chains of Urushi and the intermolecular interactions (π–π stacking) between lignin and Urushi. Additionally, in the aforementioned previously reported lignin–oleate nanoparticles, base-catalyzed cleavage of lignin–oleic ester bonds eventually disrupted the hydration barrier and destabilized the colloidal system,^37^ while such alkaline hydrolysis is not an issue with the noncovalent assemblies of Urushi-lignin hy-LNPs. Therefore, these new hy-LNPs circumvent the time-dependent destabilization of LNPs under harsh conditions.

**Figure 3:**
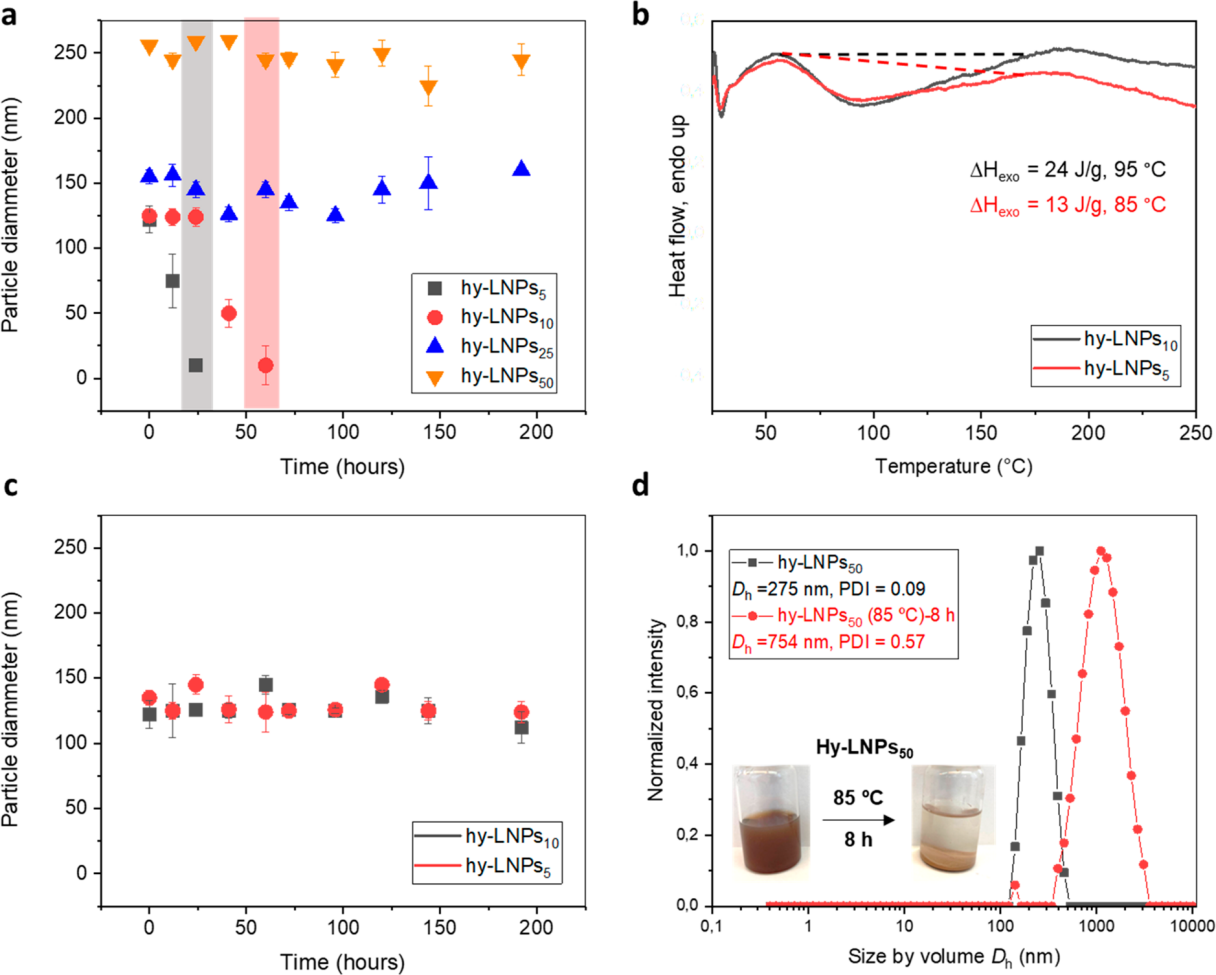
Stability of hy-LNPs in basic conditions: (a) Evolution of particle size for hy-LNPs at pH 12.0. The colored dashed sections indicate the time-dependent dissolution of particles with lower Urushi content (hy-LNPs_5_ and hy-LNPs_10_). (b) DSC thermogram corresponding to the dynamic thermal curing at 10 °C min^–1^ of freeze-dried hy-LNPs_5_ and hy-LNPs_10_. Lines indicates integration region used to determine the exothermic peak. (c) Evolution of particle size for cured hy-LNPs_5_ and hy-LNPs_10_ at pH 12.0. (d) Analysis of hydrodynamic diameter by DLS of hy-LNPs_50_ before (black traces) and after (red traces) interparticle thermal curing process. The inset corresponds to digital images of hy-LNPs_50_ colloidal dispersions before and after thermal curing.

Among the different properties of Urushi, the possibility to form cross-linked polymeric networks via thermal-induced curing processes is one of the most interesting options to form films and coatings.^40^ The curing of Urushi involves the aerobic oxidation of the unsaturated hydrocarbon chains and the subsequent radical coupling reactions.^41^ Therefore, and in order to achieve the stabilization of the hy-LNPs with lower urushi content (hy-LNPs_5_ and hy-LNPs_10_), thermal-induced internal cross-linking of those particles was explored. To find the most suitable curing temperature, thermal analysis of hy-LNPs was conducted via differential scanning calorimetry (DSC) measurements. DSC thermograms recorded from freeze-dried hy-LNPs_5_ and hy-LNPs_10_ revealed an exothermic peak associated with the coupling reactions of the unsaturated hydrocarbon chains of Urushi between 75 and 120 °C (Figure 3b). Thus, the thermal-induced curing of hy-LNPs_5_ and hy-LNPs_10_ colloidal dispersions was conducted at 85 °C for 5 h. The resulting cured particles were colloidally stable at room temperature without any sign of precipitation or aggregation for at least three months. In addition, DSC postcuring thermogram of hy-LNPs_10_ did not reveal the presence of any exothermic peak associated with the curing process, thus confirming a complete curing of the particles in the conditions employed (Figure S4). In order to study the stability of the cured hy-LNPs, kinetic experiments under harsh basic (pH > 12) conditions were employed (Figure 3c). Delightfully, the time-dependent particle size measurements of cured hy-LNPs showed only minor deviations in their particle size compared to the original ones, confirming an exceptional colloidal stability under harsh conditions owing to an efficient intraparticle cross-linking processes. We further note that when thermally induced curing was applied to hy-LNPs with a higher Urushi content (hy-LNPs_25_ and hy-LNPs_50_) a significant increase in particle size was accompanied by an aggregation and complete sedimentation of the dispersions (Figure 3d). This latter observation is attributed to interparticle cross-linking promoted by the hydrophibic Urushi chains that are present on the surface of hy-LNPs with higher content of Urushi. These results support our indications that enhanced stability is attributed to the hydration barrier effect for those particles (Figure 3a). Overall, Urushi not only emerges as a unique renewable component to achieve stabilization of LNPs via intraparticle cross-linking and hydration barrier effects, but also offers the possibility to prepare interparticle cross-linked networks that present opportunities for particulate coatings or adhesives without the use of toxic cross-linking reagents as is the case of formaldehyde,^34^ epichlorohydrin,^42,43^ or bisphenol A diglycidyl ether.^33^

Owing to the traditional use of Urushi in natural resin coatings, we decided to explore the possibility to use hy-LNPs with a higher Urushi content (hy-LNPs_50_) to prepare hydrophobic protective coating for wood via interparticle cross-linking process.^40^ In this sense, pristine birch wood was tested owing to its broad use in furniture items and construction materials. For this application, hy-LNPs_50_-coated wood specimens were obtained by deposition of hy-LNPs_50_ dispersion followed by evaporation and thermal curing process (Figure 4a). Water contact angle (WCA) measurements were assessed to study the influence of hy-LNPs_50_ coating on the hydrophobicity of wood (Figure 4b). Marked improvement in the water resistance of hy-LNPs_50_-coated wood specimens immersed in water was observed in the WCA results after short (15 s) and prolonged (24 h) immersion times compared to noncoated wood specimens, as depicted in Figure 4b. This improvement can be attributed to the hydrophobic nature of the hy-LNPs_50_ coating. These results were also supported by SEM analysis of noncoated wood and hy-LNPs_50_-coated wood specimens (Figure 4c,d). In the case of noncoated wood specimen, characteristic wood porosity can be appreciated, which allows wetting and penetration of water (Figure 4c). In contrast, in the case of hy-LNPs_50_-coated wood specimen, the presence of hy-LNPs_50_ was observed efficiently covering the wood surface without losing their spherical morphology after curing. Such a uniform coating prevents the penetration of water through the pits, thus demonstrating their potential as a hydrophobic coating to preserve wood from moisture. Last but not least, we also investigated the effect of the hy-LNPs_50_ coating on the leaching of wood components after exposing the wood to different pH (4, 7, and 10) and times (10 min, 1 h or 24 h) via optical density measurements (Figure 4e,f). Regardless of the pH buffer employed, the hy-LNPs_50_-coated wood specimen showed a markedly lower leaching of wood substances in contrast to uncoated wood (Figure 4e,f), which demonstrates the durability and versatility of the hy-LNPs_50_ coating to protect wood surface under different conditions.

**Figure 4:**
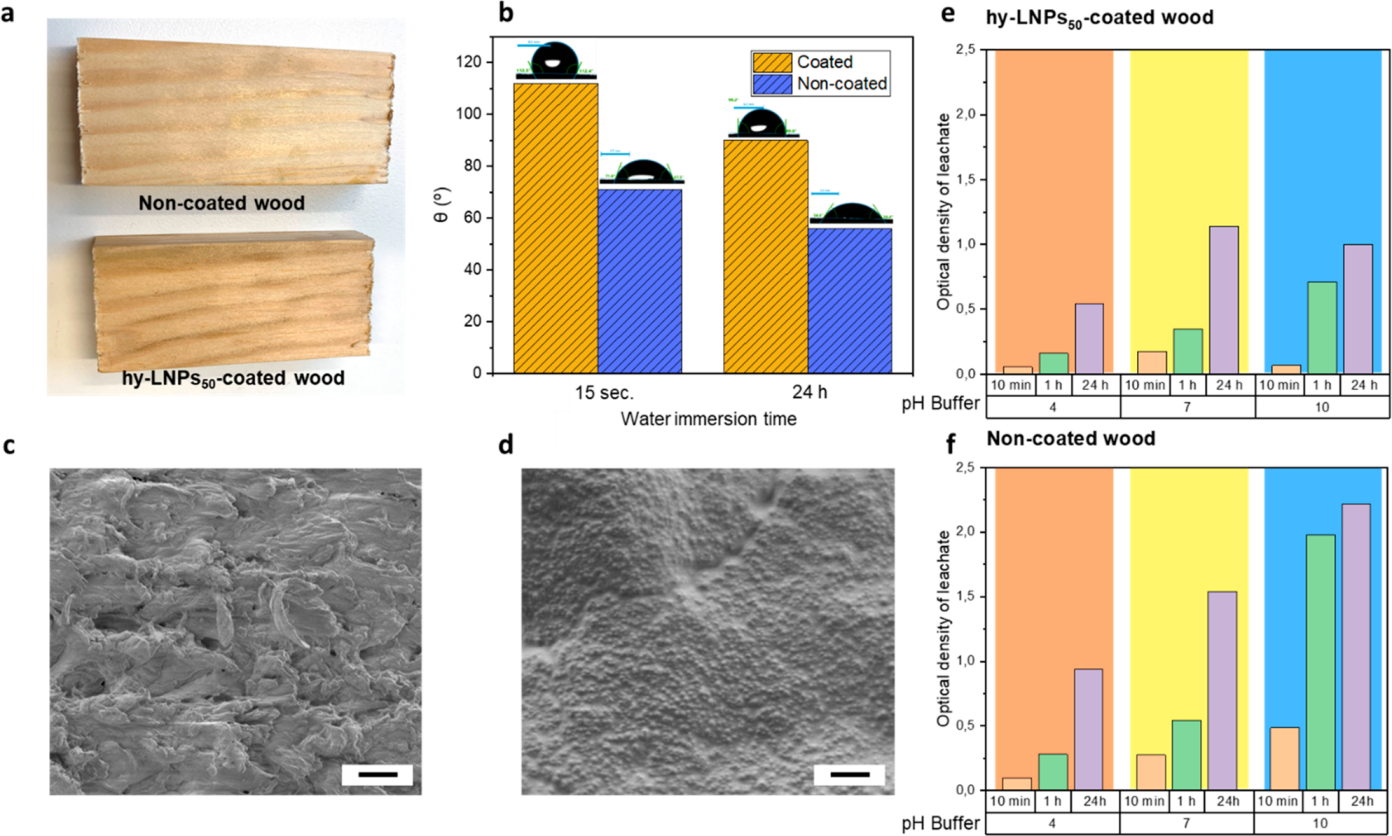
Application of hy-LNPs_50_ as hydrophobic coating for wood: (a) Digital images of noncoated and coated wood specimens used in WCA measurements. (b) Water contact angle (WCA) measurements for coated and noncoated wood specimens at different water immersion times. Scanning electron microscopy (SEM) images of (c) noncoated wood and (d) coated wood. Scale bars (1 μm). Optical density measurements of (e) coated and (f) noncoated wood specimens after exposing the wood surface to different pH buffers at different times.

In summary, we have introduced a novel technique for stabilizing LNPs either through an intraparticle covalent cross-linking process or noncovalent hydration barrier effect using Urushi as a renewable biobased component. In addition, hy-LNPs with higher amount of Urushi allow the preparation of interparticle cross-linking networks that have been demonstrated as hydrophobic coatings that increase water resistance of wood. Finally, we anticipate that the straightforward noncovalent methodology will open new avenues for the application of lignin-based particles in advanced materials, where stabilization of LNPs is needed.

## Supporting Information Available

Materials, experimental section, synthesis of materials, and additional data on characterization of hybrid lignin nanoparticles (PDF)
