# Supplementary material for: Urushi as a Green Component for Thermally Curable Colloidal Lignin Particles and Hydrophobic Coatings
Source: ACS Macro Lett. 2023 May 22;12(6):759–66. doi: 10.1021/acsmacrolett.3c00186 (PMC10286546; doi:10.1021/acsmacrolett.3c00186)
Supplement: Supplementary file 1 — mz3c00186_si_001.pdf [file mz3c00186_si_001.pdf]

Supporting Information for

Urushi as a green component for thermally curable colloidal lignin particles and hydrophobic coatings

Adrian Moreno[*1,2] *Ievgen Pylypchuk,*[1] *Yoko Okahisa*[3] *and Mika H. Sipponen*[*1,4]

[1]Department of Materials and Environmental Chemistry, Stockholm University, Svante Arrhenius väg 16C, SE-106 91 Stockholm, Sweden.

[2]Laboratory of Sustainable Polymers, Department of Analytical Chemistry and Organic Chemistry, Rovira i Virgili University, Tarragona 43007, Spain

[3] Faculty of Fiber Science and Engineering, Kyoto Institute of Technology, Matsugasaki, Sakyo-ku, Kyoto 606-8585, Japan

[4] Wallenberg Wood Science Center, Department of Materials and Environmental Chemistry, Stockholm University, SE-10691 Stockholm, Sweden

**Corresponding authors:* adrian.moreno@urv.cat; mika.sipponen@mmk.su.se

Materials

All hybrid lignin nanomaterials prepared in this work were prepared from BIOPIVA^{TM} 100 pine Kraft lignin (SKL) (UPM, Finland), previously characterized. [1] Black oriental lacquer (urushi; kuro-urushi, Kyoto, Japan) solution, manufactured in 2019, was used to prepare the hybrid particles. Ambient-dried pine wood samples. Deionized (DI) water and non-dried tetrahydrofuran was used throughout the experiments that involve particle formation. Dialysis of particles (hy-LNPs) was performed on dialysis tube benzoylated (MWCO 1000) form Merck against deionized water.

Buffers: citric acid, NaOH/HCl with pH 4 (Merck), phosphate buffer with pH 7 (VWR), boric acid/KCl/NaOH with pH 10 (Merck).

[1] Sipponen, M. H.; Farooq, M.; Koivisto, J.; Pellis, A.; Seitsonen, J.; Österberg, M. Spatially Confined Lignin Nanospheres for Biocatalytic Ester Synthesis in Aqueous Media. *Nat. Commun.* **2018**, *9*, 2300.

Methods

Transmission Electron Microscopy (TEM) images were recorded on a JEM-2100 microscope (JEOL, Japan) operating with an accelerating voltage of 200 kV. Colloidal dispersions of hy-LNPs were previously diluted by a factor of 1:40, followed by the deposition and evaporation onto a carbon coated copper grid. **Scanning Electron Microscopy (SEM)** images of non-coated wood and hy-LNPs$_{50}$-coated wood specimens with dimensions of 2x5x5 mm were recorded on a JSM-7000F (7000) FE-SEM (JEOL, Japan) operating at 5 kV. Before the analysis, the samples were vacuum dried for 3 hours at 50 ºC. **Dynamic Light Scattering (DLS)** measurements were performed at room temperature on a Zetasizer Nano ZS (Malvern, UK). The zeta potential was determined using a dip cell probe. hy-LNPs were diluted by a factor of 30 with deionized water respectively before the analysis. **Differential Scanning Calorimetry (DSC)** measurements were performed on Netzsch DSC 214 Polyma with N_2 as the purge gas (50 mL/min) and using a heating rate of 10 °C/min in the 25–250 °C temperature range. Calibration was performed using an indium standard for heat flow calibration and a zinc standard for temperature

calibration. hy-LNPs were freeze-dried before DSC curing measurements. **Water contact angle (WCA) measurements** of non-coated wood and hy-LNPs$_{50}$-coated wood specimens at different water immersion times (15 sec. and 24 h) were performed on using Contact Angle Meter model DSA25E (KRÜSS instruments, Germany). **NMR spectroscopy.** ^{1}H NMR analyses of hy-LNPs in dispersion state were performed on a 400 MHz (for ^{1}H) Varian VNMR-S400 NMR instrument at 25°C using deuterated oxide as solvent, and a NMR pulse program combining presaturation of water signal and excitation increased signal resolution developed by Pylypchuk et. al.[2] All chemical shifts are quoted on the δ scale in ppm using the residual solvent as internal standard.

[2] Pylypchuk, I. V.; Lindén, P. A.; Lindström, M. E.; Sevastyanova, O. New Insight into the Surface Structure of Lignin Nanoparticles Revealed by ^{1}H Liquid-State NMR Spectroscopy. *ACS Sustainable Chem. Eng.* **2020**, *8*, 13805-13812.

Experimental procedures

Preparation of hybrid lignin nanoparticles (UR-SKL hy-NPs). UR-SKL hy-LNPs were prepared by replacing SKL with the corresponding wt% of Urushi, but otherwise following the same procedure for the preparation of LNPs described previoulsy.[3] Briefly, Urushi and SKL were dissolved separately in THF/water mixture (mass ratio 3:1), insoluble impurities were removed by filtration and soluble fraction combined at a predetermined ratio. hy-LNPs were produced by rapid addition of deionized water to Urushi and SKL solution. After that, dispersions were concentrated by rotary evaporation and dialyzed against water for 24 h to ensure complete removal of organic solvent. The final aqueous dispersion of hy-LNPs (1 g L^{-1}) was obtained with a lignin mass yield of 71%.

[3] Moreno, A.; Liu, J.; Gueret, R.; Hadi, S. E.; Bergstrom, L.; Slabon, A.; Sipponen, M. H. Unravelling the Hydration Barrier of Lignin Oleate Nanoparticles for Acid- and Base-Catalyzed Functionalization in Dispersion State. *Angew. Chem. Int. Ed.* **2021**, *60*, 20897.

Basic stability of hy-LNPs. hy-LNPs dispersions (10 mL, 1 g L^{-1}) were adjusted to pH 12.0 by the addition of 0.7 mL of NaOH (0.1 M). Samples were incubated under stirring at room temperature. For kinetic experiments, small aliquots (0.1 mL) were withdrawn at different intervals of time to monitor de evolution of particle size by DLS.

Cross-Linking of hy-LNPs using the hydrothermal Process. A total volum of 10 mL of hy-LNPs colloidal dispersion (10, 20 or 50 wt% Urushi content) was hydrothermally cured in a 20 mL sealed vial at 85 ºC for 5 hours.

Preparation of a hydrophobic protective coating for wood via interparticle cross-linking process. 1.2 ml of particles suspension was deposited over pre-moistened wood sample (3x7 cm). After drying in air, sample was placed into the drying oven to cure for 24h at 105°C.

Wetting wood sample. Wood sample was immersed in DI water for 24h at room temperature. Then the sample was air dried at ambient conditions for 7 days.

Optical density measurements.
A wood sample with dimension 1*1*2.5 cm was submerged into 5 ml of buffer solution in a Petri dish by coated side and was left there for a certain period of time. After 10 mins, 1 hour, and 24 hours the 1 ml of the solution was transferred to the cuvette and its optical density was analyzed at λ=350 nm while using the corresponding buffer as a blank solution. The analyte solution was then poured back into a Petri dish with a wood sample.

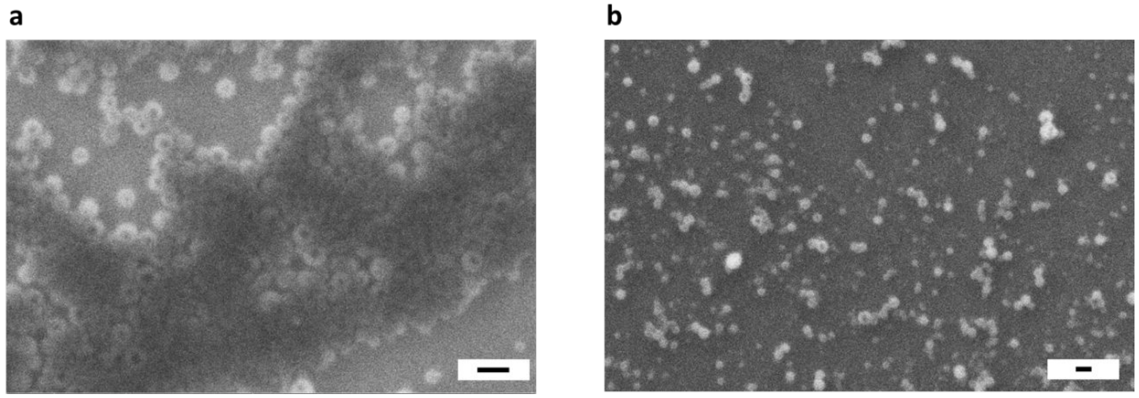

Figure S1. Scanning electron microscopy (SEM) images of (a) hy-LNPs25 and (b) hy-LNPs$_{10}$. Scale bars (250 nm).

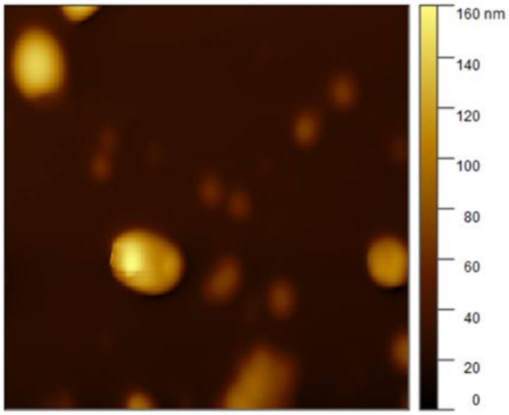

Figure S2. AFM height images of hy-LNPs$_{10}$

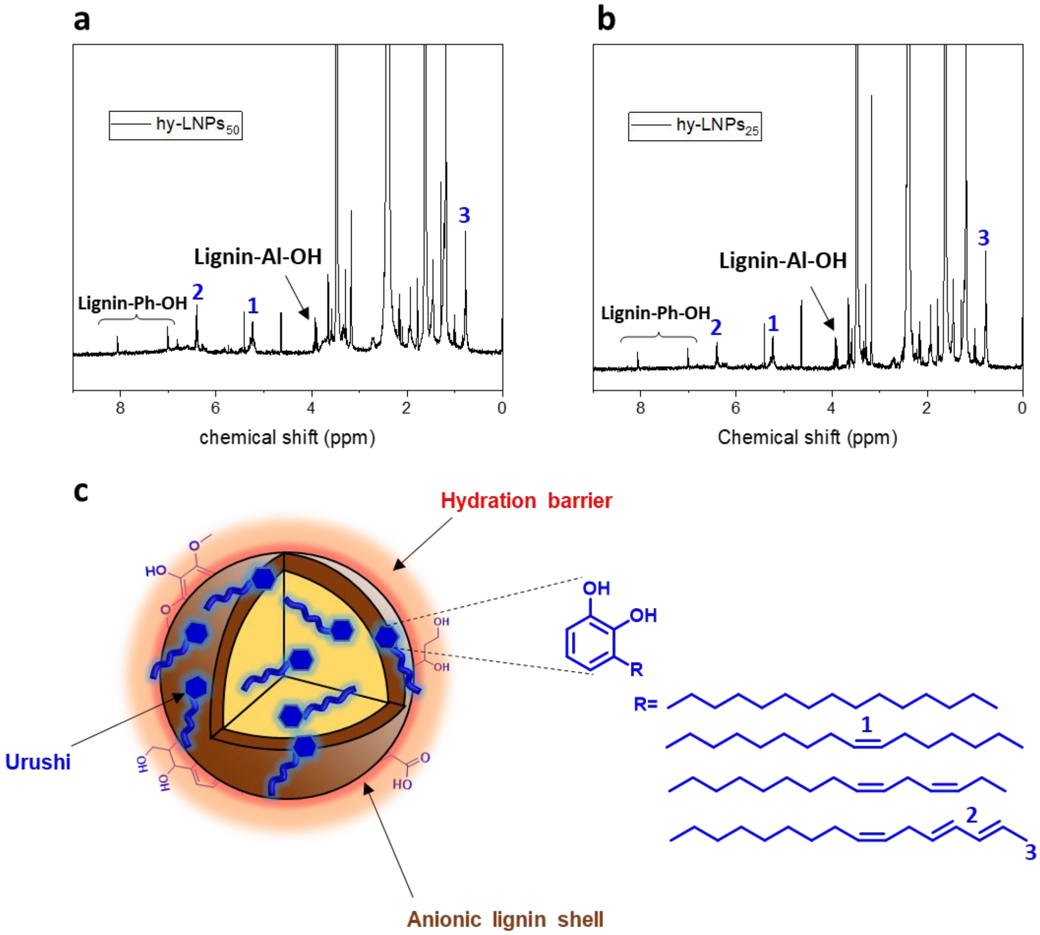

Figure S3. ^{1}H NMR spectra of (a) hy-LNPs$_{50}$ and (b) hy-LNPs$_{25}$ in dispersion state. (c) Proposed model for surface composition of hy-LNPs with high Urushi content (25 and 50 wt%).

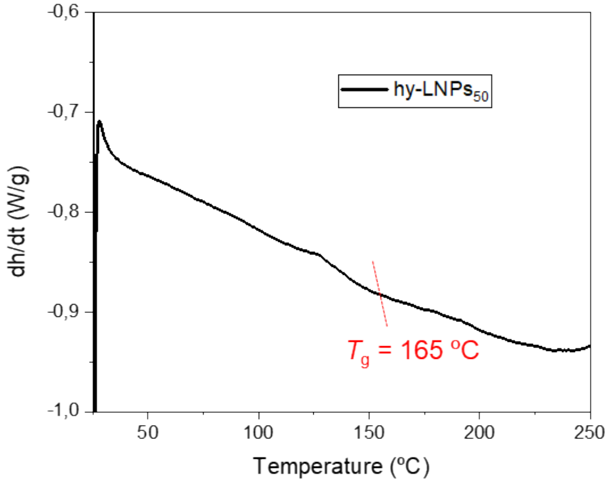

Figure S4. DSC thermogram corresponding to post curing heating process at 10 °C min^{-1} of hy-LNPs$_{10}$
